# Severe esophageal stenosis in a patient with metastatic colon cancer following palliative radiotherapy, ramucirumab, and chemotherapy

**DOI:** 10.1002/ccr3.2751

**Published:** 2020-03-09

**Authors:** Keiko Akahane, Katsuyuki Shirai, Masaru Wakatsuki, Kazunari Ogawa, Kyosuke Minato, Kohei Hamamoto, Satoru Takahashi, Koichi Suzuki, Jun Takahashi, Toshiki Rikiyama, Keita Matsumoto, Hirosato Mashima

**Affiliations:** ^1^ Department of Radiology Saitama Medical Center Jichi Medical University Saitama Japan; ^2^ Department of Radiology Jichi Medical University Shimotsuke Japan; ^3^ Department of Surgery Saitama Medical Center Jichi Medical University Saitama Japan; ^4^ Department of Gastroenterology Saitama Medical Center Jichi Medical University Saitama Japan

**Keywords:** antiangiogenic agent, bone metastasis, esophageal stenosis, palliative radiotherapy, ramucirumab

## Abstract

Antiangiogenic agents, such as ramucirumab, should be cautiously administered along with radiotherapy because of the enhanced risk of adverse events.

## Graphical abstract

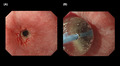

## 1 INTRODUCTION

Severe adverse events in patients who receive palliative radiotherapy during metastatic cancer treatment are rarely reported. The administration of antiangiogenic agents along with palliative radiotherapy may enhance the adverse events caused by the latter. Hence, such treatment regimens should be administered with care.

Palliative radiotherapy (RT) is widely used to alleviate symptoms, especially in patients with painful bone metastasis, and high response rates of 70%‐80% have been reported in these patients.^1^ Palliative RT is considered a safe treatment, and severe adverse events associated with palliative RT alone are rare.^2^, ^3^ The rapid advances in chemotherapy, molecular‐targeted therapy, immune checkpoint inhibitor, and antiangiogenic therapy have further improved the prognosis of patients with metastatic cancer. Antiangiogenic therapy inhibits vascular endothelial growth factor, restricting tumor angiogenesis.^4^ Although the efficacy of these agents has been reported for several cancers, severe adverse events including perforation and bleeding have been observed.^4^

Recently, the number of patients with metastatic cancer receiving antiangiogenic therapy and palliative RT has increased. However, the safety of such combination therapy has not been fully investigated. Here, we report a patient with bone metastasis from colon cancer who developed severe esophageal stenosis after receiving a combination of palliative RT and ramucirumab, an antiangiogenic agent.

## 2 CASE REPORT

**Figure 1:**
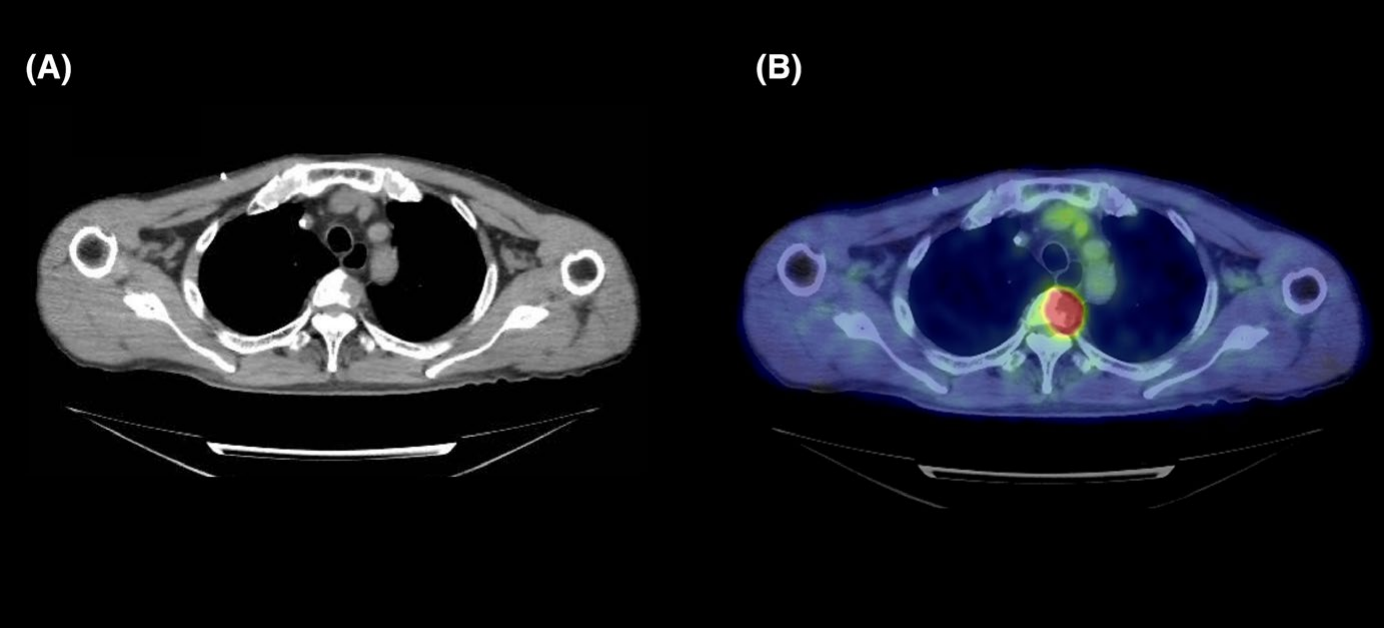
Chest plain computed tomography (CT) scan showing an osteolytic lesion on the left side of the third thoracic vertebra (A). ^18^Fluorodeoxyglucose (FDG)‐positron emission tomography (PET) scan showing high accumulation of FDG at the lesion, which was diagnosed as bone metastasis (B)

**Figure 2:**
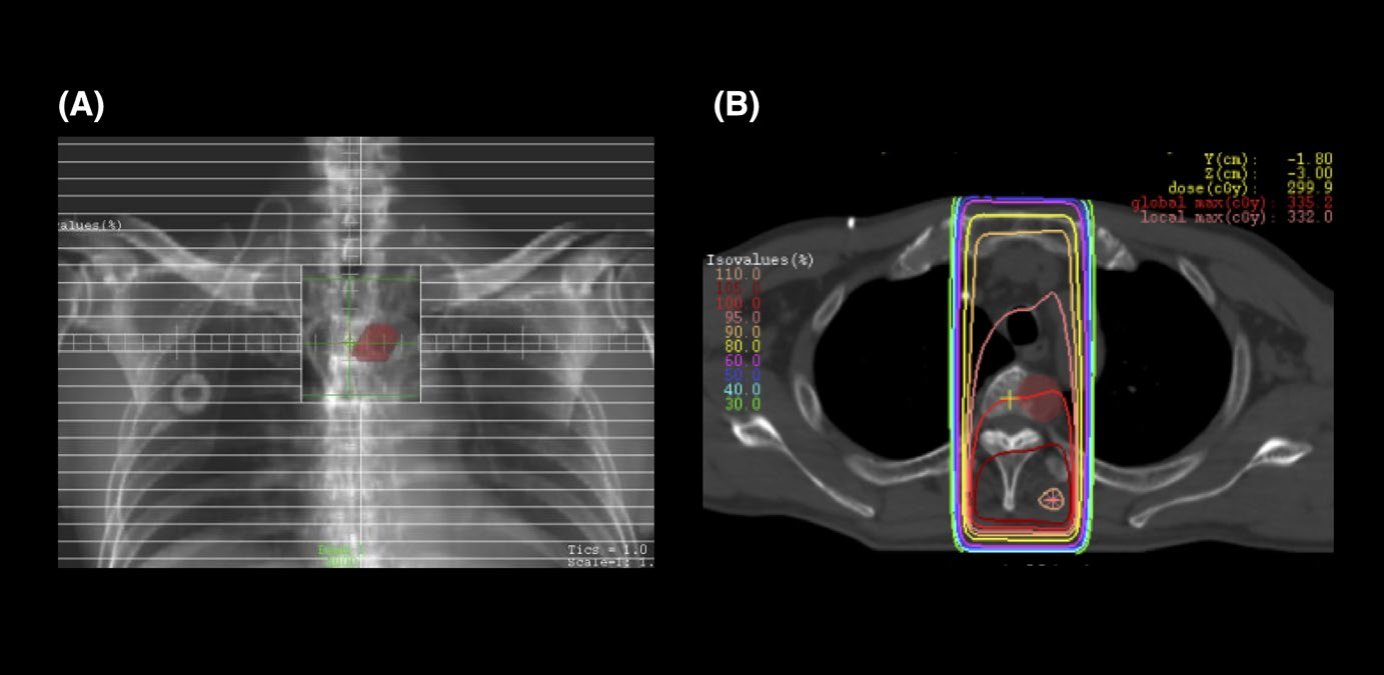
Palliative radiotherapy (RT) using 30 Gy delivered in 10 fractions for 2 wk was delivered to the third thoracic vertebra. Digital reconstructed radiography (A). Dose distribution in axial slice (B)

**Figure 3:**
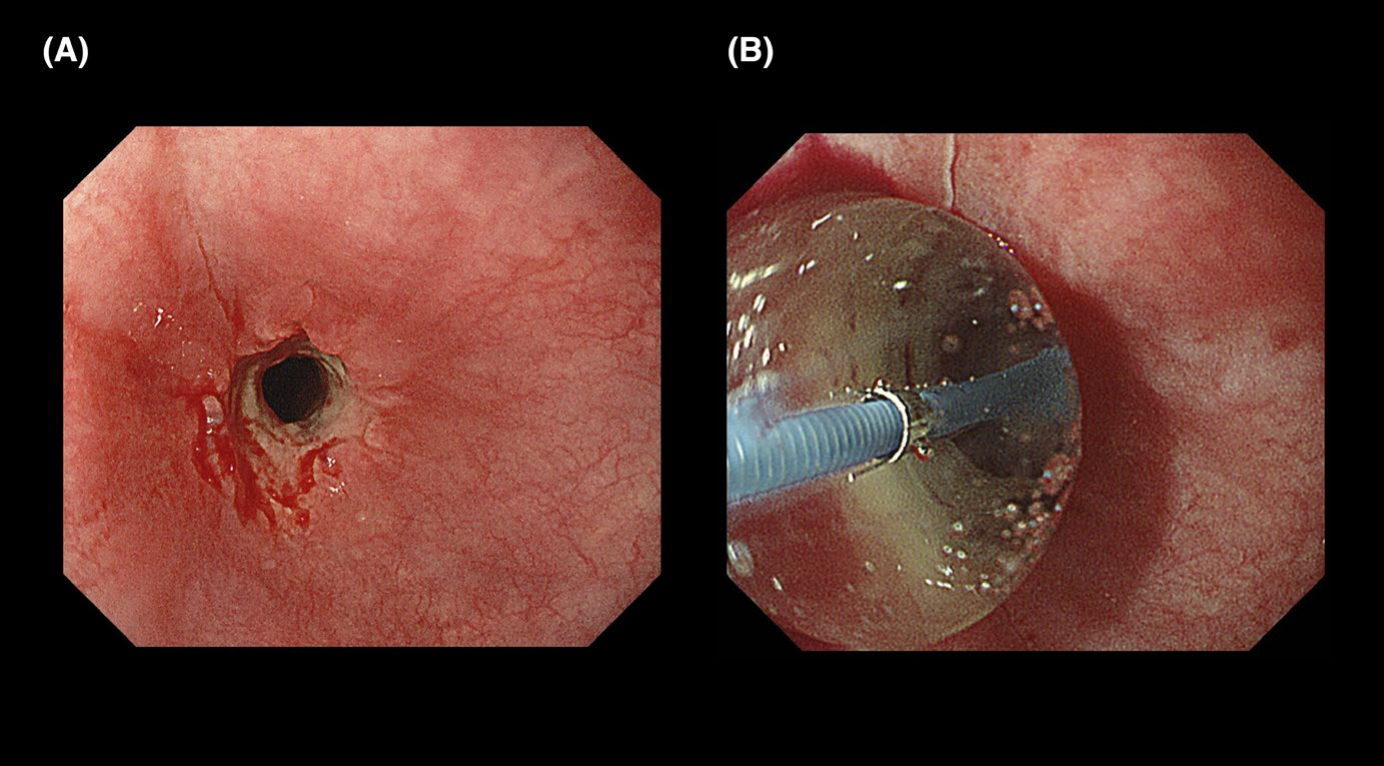
Esophageal stenosis with pseudomembrane formation and vascular ectasia was detected in the upper thoracic region of the esophagus on esophagoscopy (A). Esophagitis was also observed, and no tumor was detected. Since the stenosis was located in the irradiation field, the final diagnosis was benign esophageal stenosis related to palliative radiotherapy (RT). Balloon dilation with a diameter of 12 mm was performed with little bleeding and no complications (B)

A 65‐year‐old man was diagnosed with colon cancer with metastasis to the liver. The histopathological diagnosis was well‐differentiated adenocarcinoma (KRAS mutation‐positive), and the clinical stage was T2N1M1. The patient did not have diabetes mellitus, collagen disease, or reflux esophagitis. He received six courses of FOLFOX (85 mg/m^2^ intravenous oxaliplatin, 200 mg/m^2^ intravenous leucovorin, and 400 mg/m^2^ intravenous fluorouracil bolus then 2400 mg/m^2^ continuous infusion) with bevacizumab (5 mg/kg intravenous infusion) and seven courses of FOLFOX without bevacizumab. Two months after completion of the chemotherapy, the patient underwent palliative RT (30 Gy in 10 fractions for 2 weeks) using an anterior‐posterior/posterior‐anterior RT field to treat a metastatic lesion in the third thoracic vertebra detected on ^18^fluorodeoxyglucose‐positron emission tomography/computed tomography (CT) (Figures 1 and 2). Ramucirumab (8 mg/kg intravenous infusion) and FOLFIRI (150 mg/m^2^ intravenous irinotecan, 200 mg/m^2^ intravenous leucovorin, and 400 mg/m^2^ intravenous fluorouracil bolus then 2400 mg/m^2^ continuous infusion) were administered after the patient had received the 9th fraction of palliative RT. The patient developed dysphagia following treatment, complaining of severe pain on swallowing, and requiring a liquid diet and analgesics. Esophagoscopy was performed 6 weeks after the completion of palliative RT to determine the cause of persistent dysphagia in the patient. Severe esophageal stenosis was detected in the upper thoracic region of the esophagus within the RT field (Figure 3A). Balloon dilation was performed in the hospital (Figure 3B), and the symptoms of dysphagia rapidly improved. This adverse effect was considered acute esophageal stenosis grade 3, according to the Common Terminology Criteria for Adverse Events, version 5.0. There were no other adverse events, such as radiation dermatitis, bronchitis, pneumonitis, and radiation myelopathy. FOLFIRI and ramucirumab were reintroduced 2 weeks after balloon dilation of the esophagus. The patient did not experience a relapse of the dysphagia or complain of any other esophageal symptoms until his death.

## 3 DISCUSSION

In this case, palliative RT and ramucirumab led to the development of severe esophageal stenosis in a patient with vertebral metastasis of colon cancer. Bone metastases are frequently observed in advanced cases of colon cancer, and the spine is the most common metastatic site.^5^ RT is an effective therapy to palliate pain and improve the quality of life in patients with bone metastases.

Esophageal stenosis is a rare adverse event associated with RT. Generally, RT temporarily induces acute esophagitis, dysphagia, and pain on swallowing, which can be treated by conservative therapy including mucosal protectant and analgesics. In most cases, these symptoms improve immediately after the completion of RT, but some patients may develop esophageal stenosis or ulcers. High radiation doses with concurrent chemotherapy have been reported as risk factors for severe esophageal toxicity.^6^

Radiation‐induced esophageal stenosis is considered to be due to chronic inflammation and fibrosis caused by RT,^7^, ^8^ which can be safely and effectively treated with balloon dilation.^9^ The incidence of esophageal stenosis is reportedly 6% in patients with lung cancer who have received concurrent chemoradiotherapy.^10^ However, to the best of our knowledge, our report is the first to reveal that the low dose of radiation in palliative RT may induce esophageal stenosis when combined with ramucirumab and other chemotherapeutic regimens.

Ramucirumab, an antiangiogenic agent, is a human immunoglobulin G1 monoclonal antibody receptor antagonist of vascular endothelial growth factor receptor 2.^11^ Several phase III studies have shown that the addition of ramucirumab to a chemotherapy regimen improves the overall survival of patients with gastric cancer, gastroesophageal junction adenocarcinoma, and colorectal cancers.^12^, ^13^, ^14^ Severe adverse events related to vascular endothelial growth factor pathways such as bowel perforation and bleeding have been reported.^15^ The safety of the combination of ramucirumab and palliative RT remains unclear because these phase III studies excluded patients receiving RT. Recently, a life‐threatening tracheoesophageal fistula was observed in a patient with lung cancer after receiving ramucirumab along with radical chemoradiotherapy (60 Gy).^16^ The study used a high radiation dose of 60 Gy, which can cause a more severe adverse effect on the esophagus than that in our study using 30 Gy. Given these results, we believe that ramucirumab has the potential to enhance the adverse effects of RT.

In our study, FOLFIRI (leucovorin, fluorouracil, and irinotecan) also comprised the treatment regimen along with palliative RT and ramucirumab; hence, FOLFIRI, alone or in combination with ramucirumab, may also have affected the esophageal adverse events induced by RT. The tolerability and toxicity of FOLFIRI and RT remain unknown, but RT and FOLFOX (leucovorin, fluorouracil, and oxaliplatin), which comprises a combination of drugs similar to that included in FOLFIRI, is reportedly a safe adjuvant therapy for patients with rectal cancer.^17^ Therefore, we believe that ramucirumab is the radiation‐sensitizing agent associated with esophageal stenosis in this case.

Adverse events of the combination of other antiangiogenic agents and RT have been indicated in a few studies. Phase II studies using bevacizumab and concurrent chemoradiotherapy for patients with lung cancer were terminated early after tracheoesophageal fistulae were often observed in these patients.^18^ Findings from these studies indicate that the addition of an antiangiogenic agent to the chemoradiotherapy regimen may cause more adverse effects compared with that caused by chemoradiotherapy alone. Adverse events induced by bevacizumab, such as tracheoesophageal fistulae, were even reported 20‐21 months after chemoradiotherapy in patients with lung cancer, making long‐term follow‐up periods in these patients necessary.^19^, ^20^ The authors indicated that even a history of mediastinal RT using high‐dose radiation may be a risk factor for the development of life‐threatening fistulae upon receiving bevacizumab. Generally, the combination of chemotherapy with palliative RT is considered relatively less risky because the radiation dose in palliative RT is lower than that in radical RT. However, life‐threatening bowel perforation has been recently reported in a patient with renal cell carcinoma after administering 8 Gy of palliative RT and sorafenib, which has antiangiogenic activity.^21^ Therefore, the combination of palliative RT and antiangiogenic therapy should be administered with care, even if the radiation dose is relatively low. High‐precision RT, including intensity‐modulated RT and stereotactic RT, may decrease the adverse effects on normal tissues. Currently, our institutional policy is to avoid administering antiangiogenic therapy a week before initiating palliative RT and a week after the last fraction of palliative RT has been administered. Furthermore, patients should be monitored closely for any adverse events associated with palliative RT if antiangiogenic agents are administered at any time after palliative RT. Multi‐institutional retrospective studies are needed to evaluate the safety of the combination of any antiangiogenic agent and palliative RT.

## CONFLICT OF INTEREST

The authors state that they have no conflict of interest.

## AUTHORS’ CONTRIBUTIONS

A K, Shirai K, and Wakatsuki M: designed the analysis. Akahane K, Ogawa K, Minato K, Hamamoto K, and Takahashi S: contributed to the analysis of the results. Suzuki K, Takahashi J, and Matsumoto K: performed data collection. Shirai K, Rikiyama T, and Mashima H: supervised the project. All authors approved the final manuscript.
